# “May I Grab Your Attention?”: An Investigation Into Infants' Visual Preferences for Handled Objects Using Lookit as an Online Platform for Data Collection

**DOI:** 10.3389/fpsyg.2021.733218

**Published:** 2021-09-10

**Authors:** Christian M. Nelson, Lisa M. Oakes

**Affiliations:** Department of Psychology and the Center for Mind and Brain, University of California, Davis, Davis, CA, United States

**Keywords:** infant, visual preference, motor development, online testing, Lookit

## Abstract

We examined the relation between 4- to 12-month-old infants' (*N* = 107) motor development and visual preference for handled or non-handled objects, using Lookit (lookit.mit.edu) as an online tool for data collection. Infants viewed eight pairs of objects, and their looking was recorded using their own webcam. Each pair contained one item with an easily graspable “handle-like” region and one without. Infants' duration of looking at each item was coded from the recordings, allowing us to evaluate their preference for the handled item. In addition, parents reported on their infants' motor behavior in the previous week. Overall, infants looked longer to handled items than non-handled items. Additionally, by examining the duration of infants' individual looks, we show that differences in infants' interest in the handled items varied both by infants' motor level and across the course of the 8-s trials. These findings confirm infant visual preferences can be successfully measured using Lookit and that motor development is related to infants' visual preferences for items with a graspable, handle-like region. The relative roles of age and motor development are discussed.

As infants achieve motor milestones, they gain access to new information about the objects around them. Infants who sit up can pick up objects and look at them from many angles, and infants who crawl can see and move to objects in the distance. Thus, changes in infants' bodies and motor abilities help determine what information they have access to, attend to, and learn about (Kretch et al., 2014; Smith et al., 2018). For example, the emergence of sitting and changes in object manipulation are associated with infants' 3-D object completion abilities (Soska et al., 2010), attention to object features in dynamic events (Perone et al., 2008; Baumgartner and Oakes, 2013), and figure-ground segregation (Ross-Sheehy et al., 2016). Moreover, experience reaching and grasping objects with “sticky mittens” can induce changes in infants' attention to objects (Needham et al., 2002), interest in faces (Libertus and Needham, 2011), and perception of causal interactions (Rakison and Krogh, 2012). Shifts from crawling to standing and walking are associated with mental rotation ability (Frick and Möhring, 2013) and changes in how infants initiate eye contact with caregivers (Yamamoto et al., 2019). The emergence of walking comes with even more variation in experience with distal objects in the environment (Karasik et al., 2011). Taken together, it is clear that motor achievements in the first year have cascading effects on other aspects of development (Oakes and Rakison, 2019).

In the present investigation, we ask how infants' visual preference for items with a more easily graspable region is related to changes in their motor abilities. We focused on potentially graspable objects because Corbetta et al. (2018) describe a perception-action loop by which infants reach for objects they see, inducing changes in their reaching, manual exploration, and visual inspection of those objects. Additionally, Libertus et al. (2013) found that infants with more reaching experience shift from an initial preference for larger, more salient objects toward studying the features of smaller, more graspable objects. In the current experiment, we included a wide range of developmental achievements—examining the preference for potentially graspable objects in a sample spanning pre-sitting to walking infants—to capture changes in visual perception from the cascading effect of motor development across infancy (Oakes and Rakison, 2019; Iverson, 2021).

A secondary goal of this study was to demonstrate the effectiveness of answering such questions using methods designed to assess infant cognition while physically distant. The COVID-19 pandemic introduced unique challenges to studying infant development while physically distant. Methods of examining visual preferences at a distance may provide opportunities to study infant development even beyond the pandemic. The adoption of such tools will diversify developmental science by removing barriers to participate that have been a limitation of traditional methods.

We used Lookit, which was developed to assess visual preference using participants' own computer, monitor, and web camera (Scott and Schulz, 2017). Our goal was to examine infants' visual preference for handled objects over non-handled objects. In addition, we asked parents to report on their infants' sitting, crawling, standing, and walking in the previous week, so we could determine whether infants' preferences for handled objects in our task was related to motor development. We chose these milestones because: (1) Sitting has been associated with increases in exploratory behaviors (Soska and Adolph, 2014), better prehensile hand use (Rochat and Goubet, 1995), and looking preferences for graspable objects (Libertus et al., 2013); (2) Crawling experience has been associated with infant visual perception of objects (Cicchino and Rakison, 2008; Schwarzer et al., 2013; Gerhard and Schwarzer, 2018); and (3) Standing and walking are associated with changes in visual input (Libertus and Hauf, 2017; Franchak, 2018) and visual perception (Frick and Möhring, 2013).

## Method

### Participants

To be eligible, infants must be born at term and residing in the US. We collected data between 08/26/2020 and 01/26/2021, until we had recorded 159 sessions, anticipating a moderate effect size (e.g., ~0.5 cohen's *d*) and that we may be unable to use half of the data collected.

We excluded 30 sessions because the infants were ineligible; the infant did not reside within the United States (*n* = 6), was premature (*n* = 13), participated multiple times (*n* = 7), or was outside our target age range (*n* = 4). We excluded an additional 24 sessions because of technical problems (e.g., no video data, slow upload speeds, *n* = 15), other problems or distractions (e.g., parent peeking throughout the session, infant's eyes not visible, *n* = 6), or lack of infant interest (see data processing below, *n* = 1). Our final sample was 107 infants (*M* age = 248.30 days, *SD* = 70.26, 39 girls; histogram of age distribution is in Supplemental Materials).

Infants were recruited via the Lookit recruiter (i.e., emails were sent to families with accounts in Lookit), social media (ads on Facebook), and emails to families who had expressed interest in participation (see Oakes et al., 2021 for details regarding identification and recruitment of infant participants). All families residing in the US received a $5 Amazon gift card^1^.

Our sample was racially diverse and highly educated. Of our 107 infants, 57 were White, one was Black/African American, 10 were Asian American, 38 were multiracial, and one was unreported. Regardless of race, 19 infants were Hispanic. One (or both) parents had at least some college in 103 of the families, neither parent had any college in three families, and one family declined to state parental education. Ninety-eight families reported income; 58 reported income over $100,000, 29 reported income between $50,000 and $100,000, and 11 reported income less than $50,000.

### Stimuli

**Figure 1:**
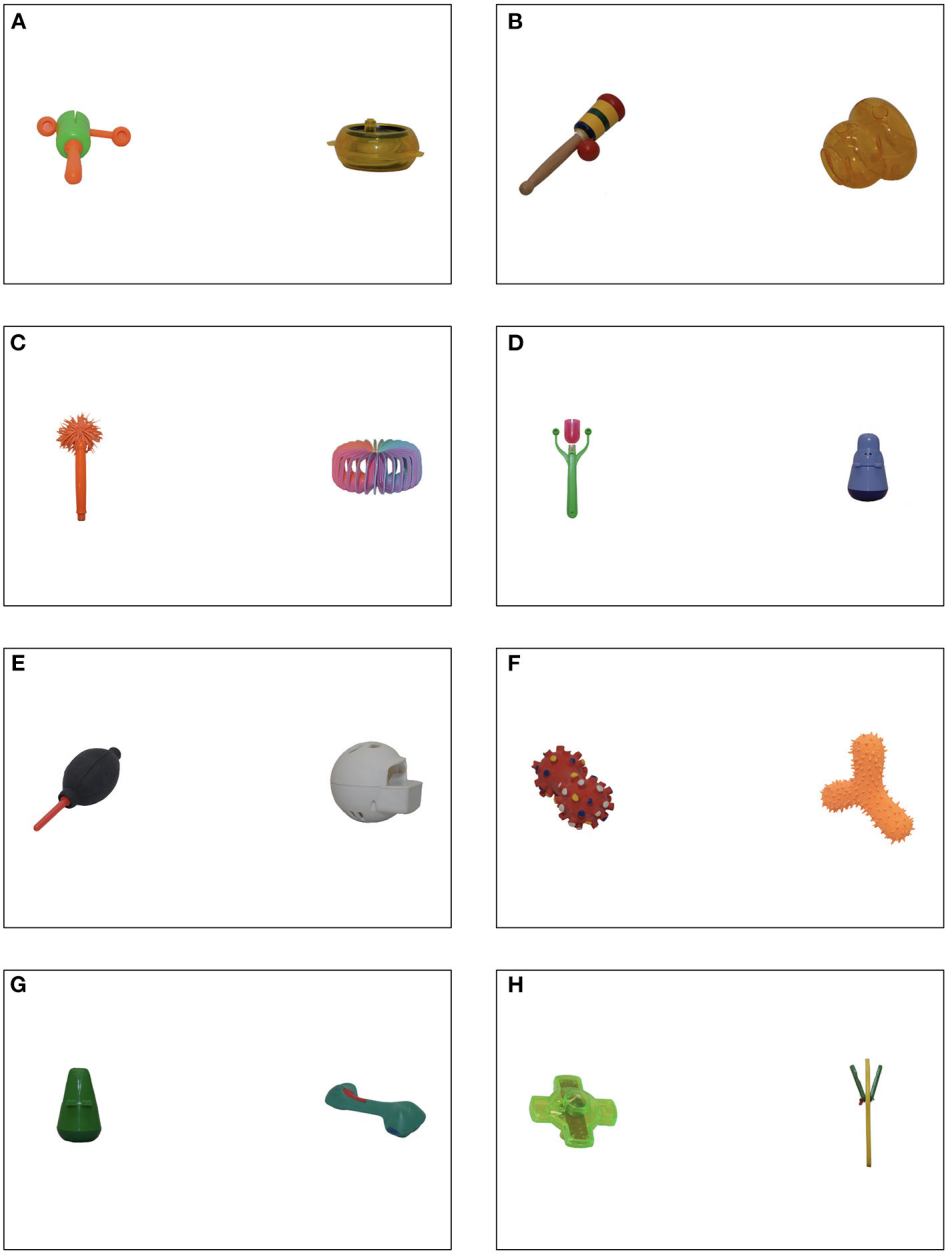
The pairs of objects used in the study. Pairs **(A)** through **(E)** show the handled object on the left. Pairs **(F)** through **(H)** show the handled object on the right. Objects came from Horst and Hout (2016).

Stimuli were photographs of 16 real, unfamiliar objects selected from the NOUN database (Horst and Hout, 2016), eight with handle-like protrusions (see Figure 1). Because the objects were novel and the handle-like protrusions varied, any preference for the handled objects would be the result of the infants' perception of the difference between the two types of objects, and not related to their knowledge of or experience with those items.

Because the Lookit platform involves each family using their own computer and monitor for the test, precise measurements of the stimuli as they were shown to each infant are not possible. However, each stimulus occupied ~5% of the total display, and were separated by a distance that was ~50% of the display. Comparison of the proportion covered by handled (*M* = 5.23%, *SD* = 2.78%) and non-handled (*M* = 4.72%, *SD* = 1.78%) objects revealed that, on average, there were no significant differences in the sizes of the two sets of objects, *t*_(14)_ = 0.44.

The objects differed on many dimensions (e.g., color, presence of pattern). To ensure that the objects did not differ in physical salience, we calculated the physical salience of each object in each stimulus pair using the Graph Based Visual Salience toolbox (GBVS, Harel et al., 2007), using the default settings. We used the GBVS toolbox because other research suggested it best predicts infant looking (Pomaranski et al., in press). We averaged the salience for the pixels in areas of interest—a region in the display that contained the objects on each stimulus. Thus, our salience values reflect the average salience of these regions for each of the pairs in our stimuli. Comparison of the average salience level for handled and non-handled objects revealed no difference in salience, *t*_(14)_ = 1.46, *p* = 0.17.

### Procedure

All sessions were conducted online, using Lookit. Parents created an account on Lookit, at which time they provide demographic information (e.g., state of residence, infant race, parent education). When ready to participate, parents logged into Lookit and selected our study. First, parents watched a short video describing our study. Then they read the consent document and verbally consented via video recording, as required on the Lookit platform. Next, parents reported whether their infant had exhibited five different poses or behaviors in the previous week; each question was accompanied by images taken from the Alberta Infant Motor Scale (Piper et al., 1992) to depict motor milestones. Parents answered, “Yes”, “No” or “Unsure” regarding whether in the past week their infant (1) sat, (2) crawled (on belly or on hands and knees), (3) pulled to stand or (4) walked independently. For scoring, we determined the highest level that the parent said “yes” to (even if the previous levels were “no” or “unsure”), and classified infants as pre-sitters (score of 1, if parents said no or unsure to all of the behaviors), sitters (score of 2, if parents said “yes” to sitting, but not to crawling, standing, or walking), crawlers (score of 3, if parents said “yes” to crawling, but not to standing or walking), standers (score of 4, if parents said “yes” to standing but not walking), or walkers (score of 5, if parents said “yes” to walking). Note that infants could “skip” a motor milestone (e.g., pull to stand or walk without crawling). We focused only on the highest motor milestone achieved, regardless of whether earlier milestones were skipped. Finally, parents viewed an instructional video illustrating how to hold their infant on their lap, facing the computer monitor (i.e., acting as a good “chair” for their infant), keeping their eyes closed during the session.

**Figure 2:**
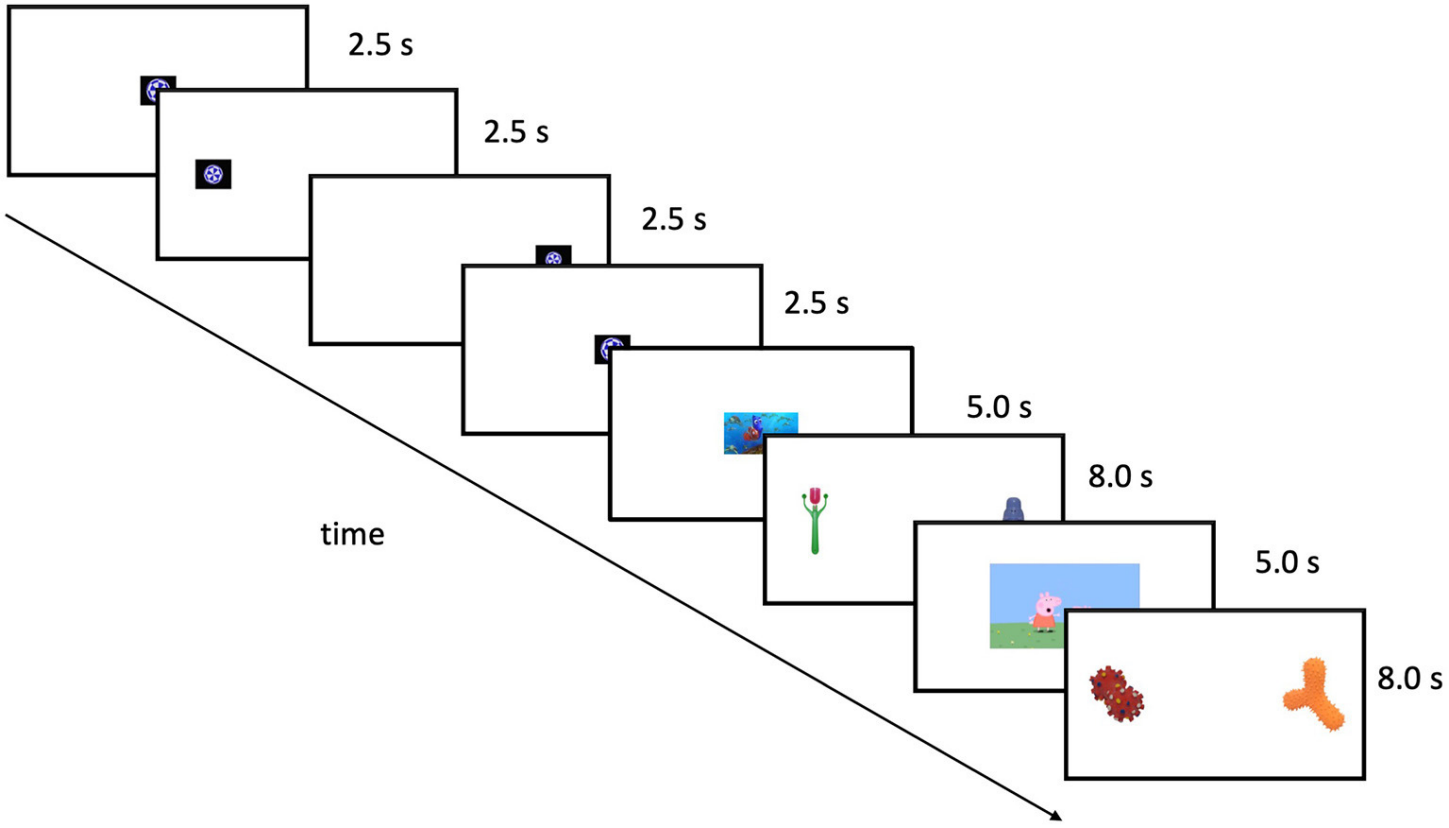
A schematic illustration of the sequence and timing of events during the experiment. Objects came from Horst and Hout (2016).

When ready, parents began the experimental session by pressing a key on their computer keyboard, which initiated a sequence of trials that continued without interruption (see Figure 2), unless the parent pressed the spacebar to pause (paused trials were excluded from the analyses). Each trial began with a 5-s attention-getting stimulus (i.e., a clip from an animated children's movie or television show) presented at the center of the display. The experiment consisted of two trial blocks. The first trial of each block was a *calibration trial*, in which a looming object, accompanied by a jingling bell, appeared for 2.5-s first at the center, then to the left, then to the right, and finally to the center again. During these trials, at any given moment there was only one item present, directing infants' attention to each location and allowing coders to calibrate their judgments about infant looks to the left or the right. After the calibration trials, there were four 8-s paired preference trials, each presenting a single pair of objects (one handled and one non-handled) accompanied by classical music. All infants saw the same eight pairs, which were divided into two blocks (A and B); within each block, the stimuli were presented in a random order for each infant. Twenty-seven infants received block A first followed by block B, and 80 infants received block B first. This uneven distribution resulted from us using one order for the first weeks of data collection and then switching the order. Because the trials are ordered randomly within blocks, this unbalanced design will have minimal impact on the results.

### Coding

Trained undergraduate research assistants used Datavyu (*https://datavyu.org/*) to code infants' looking on all trials. Because during the calibration trials there was only one stimulus presented at a time, the infants' looking to the left or right on was less ambiguous than during the experimental trials when two stimuli were presented side-by-side. Coders used the calibration trials to provide clear examples of the particular infants' left and right looks. Coders then viewed the paired preference trials; first they viewed the trials in real time and then on a second pass they used the Datavyu jog function to identify the start and end of each look to the left, right, or center (e.g., to the attention-getter at the start of the trial). A “look” consisted of at least three consecutive frames of gaze to the same location. The primary coder recorded looking on all trials, and these data were used in our analysis. A second reliability coder recorded the looking on two randomly selected test trials for each infant included in our final sample. The frame-by-frame agreement between the two coders was, on average, 94% (range: 75% to 99%−103 were above 80%).

### Data Processing

We calculated infants' total looking to the handled and non-handled object on each trial (number of frames directed to each side X frame duration^2^) and the duration of each individual look, which was defined as any successive frames to the same region (left, right, or center). We calculated handle preference scores for the trial as a whole by dividing the infants' looking to the handled object by their total looking to the two objects combined.

After participant exclusions, we evaluated 817 trials from 108 infants. We excluded 96 trials because the infant was fussy, failed to look, or their eyes were not visible; the parent looked at the experimental stimuli (a brief peek of no more than 1 s was allowed); or distraction (e.g. someone talked to and/or touched the infant, background noise). One infant was removed at this stage because they looked <2,000 ms on all trials. Thus, our analyses were conducted on 721 trials from our final sample of 107 infants.

### Analysis Plan

All analyses were conducted in R (R Core Team, 2019). To provide an *overall* impression of the data, we calculated a single score for each infant by averaging their preference scores across trials. To understand how infants' preference changed over time, we examined *trial-level* and *look-level* behavior with linear mixed-effects models, using the packages *lme4* (Bates et al., 2015) and *lmerTest* (Kuznetsova et al., 2017). Omnibus *F*-statistics were used to evaluate the significance of the fixed effects from these models, and the *emmeans* package (Lenth et al., 2018) to extract marginal means from the omnibus F-test. These two models will allow us to determine how infants' interest in the handled item relative to the non-handled item changed over time and trials, controlling for relative size and salience of the handled item on each trial. For each model, we first assessed the multicollinearity of the variables using the package *performance* (Lüdecke et al., 2021). Because we had no predictions related to infant sex, we did not include infant sex as a factor in our models. However, for transparency and consistency with NIH guidelines regarding reporting of sex as a biological factor, we disaggregate by sex when graphing our results.

To examine infants' preference at the level of trial, we conducted an analysis with handle preference on each trial as the DV (handle preference was centered by subtracting chance, or 0.50, for ease of interpretation). We included fixed effects of motor level, trial number, and the interaction between these variables. We also included control variables of relative salience and relative size of the handled item. We included random effects of child and stimulus (i.e., the unique object pairs presented on each trial). An initial model revealed collinearity between age in days and motor level. Thus, our final model included only motor level, which was our variable of interest.

Finally, we conducted a model with the duration of each individual look as DV. We included fixed effects of motor level, object type (handled or not), look index (e.g., whether it was the first look, second look, and so on), and interactions between these variables. We also included control variables of stimulus salience, and stimulus size, and random effects of child and stimulus. Again, an initial model revealed that age in days and motor level were collinear, so our final model included motor level.

## Results

Infants contributed on average 6.74 trials (*SD* = 1.74, range 1 to 8) to the analysis and looked on average 5079.3 ms (*SD* = 1084.86 ms) on each trial. Infants' age in days was not significantly correlated with average duration of looking, *r*(107) = 0.004, or the number of trials completed, *r*(107) = 0.01. Motor level also was not significantly correlated with average duration of looking, *rs*^3^ (107) = 0.09, or with the number of trials completed, *rs* (107) = 0.11. Unsurprisingly, motor score was significantly correlated with infants' age in days, *rs* (107) = 0.87, reflecting the fact that older infants were more motorically advanced than younger infants.

Our first analyses examined infants' overall handle preference, both averaged across all completed trials and examining preferences trial by trial. The average preference score for the group of infants as a whole was.54 (*SD* = 0.10), which was significantly greater than chance (0.50), *t*_(106)_ = 4.23, *p* < 0.001, *d* = 0.41. Planned comparisons conducted for each motor group revealed that only the locomotor infants (crawlers, standers, and walkers) had handle preferences that were significantly greater than chance (see Table 1), however motor level and handle preference were not significantly related, *rs* (107) = 0.12.

**Table 1:** Mean handle preference and age (in days) by Motor Level.

**Motor Level**	***N***	**Mean Age**	**Mean handle preference**	***t***	***p***	***d***	**Scaled JZS Bayes Factor**
Pre-sit	15	149.67(*sd* = 26.14, 124–212)	0.53	1.14	0.27	0.29	BF_01_ = 2.19
Sit	18	205.94(*sd* = 41.88,160–285)	0.53	1.23	0.23	0.29	BF_01_ = 2.14
Crawl	21	211.67(*sd* = 33.36,124–259)	0.55	2.38	0.03	0.52	BF_10_ = 2.21
Stand	44	295.20(*sd* = 36.34,206–362)	0.54	2.12	0.04	0.32	BF_10_ = 1.24
Walk	9	353.36(*sd* = 15.26,328–375)	0.61	3.74	0.01	1.25	BF_10_ = 9.95

*t-tests are one-sample t-tests comparing the means to chance (0.50). The Bayes Factors were calculated using the non-informative JZS prior with a scale factor of 0.707. BF_10_ indicates support for the alternative hypothesis and BF_01_ indicates support for the null hypothesis; BF between 1 and 3 provides anecdotal evidence, and BF between 3 and 10 provides moderate evidence*.

We also conducted the LMM on infants' overall preference on each trial as specified earlier. This model revealed no significant effects of interactions. We conducted an analysis with age instead of motor level, which also did not reveal any significant effects or interactions (see Supplementary Materials).

**Figure 3:**
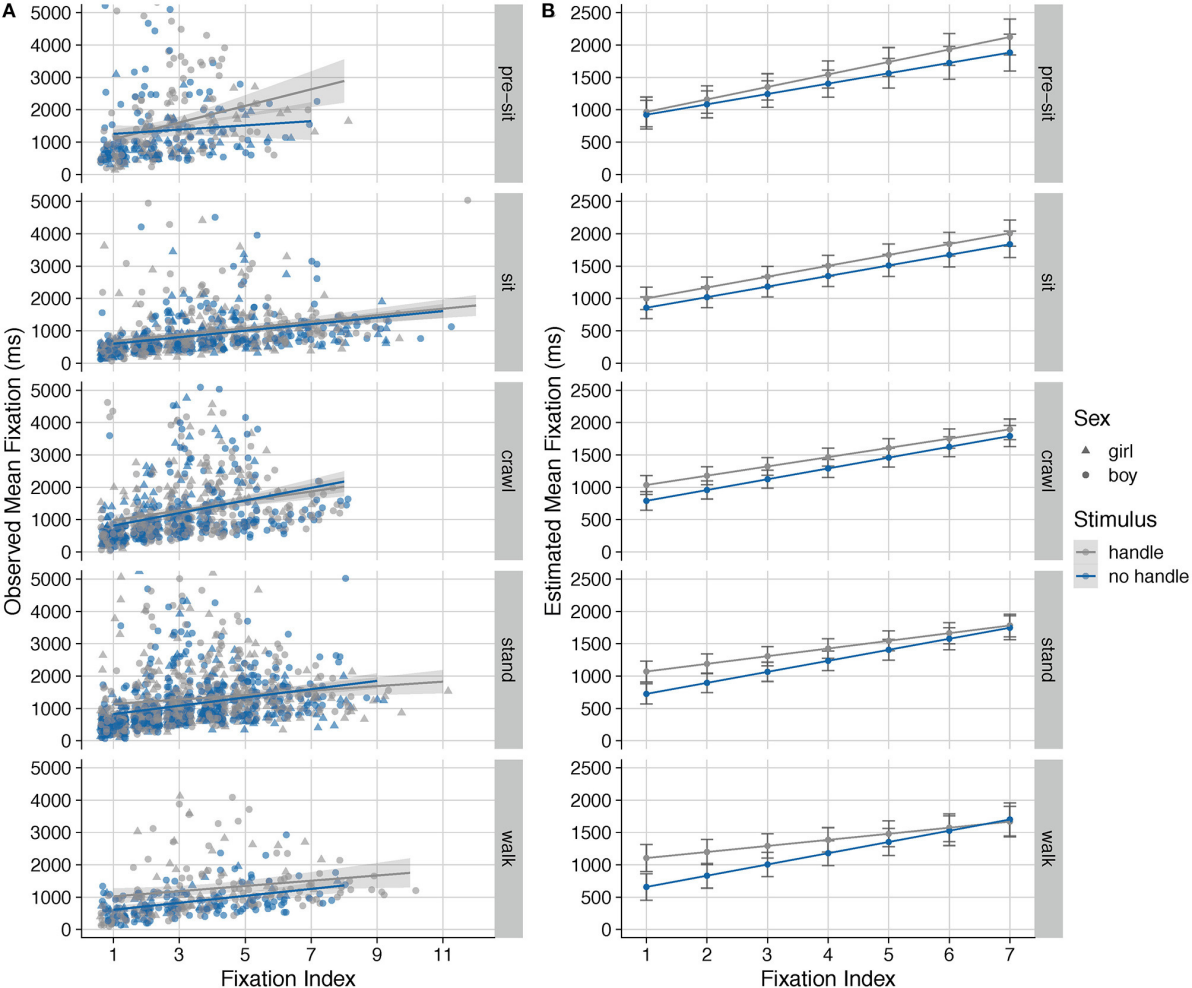
The observed data points (one point for each look for each infant) and illustrative regression lines **(A)** and the estimated marginal means **(B)** for the duration of individual looks (in ms) to handled (pink) and non-handled (blue) objects, separately for each motor level. The figure for the observed data **(A)** has been artificially cut off at 5,000 ms, which means that 43 looks of longer duration are not represented on the figure. The marginal means have been estimated only for the first seven fixations, as the observed data are sparse for later fixations and the estimated means will be unreliable. The error bars in the marginal means represent 95% confidence intervals. Objects came from Horst and Hout (2016).

Finally, we examined the duration of each individual look during a trial. Infants contributed, on average, 4.48 looks (*SD* = 1.34, range 1 to 12). On average looks to the handled objects were longer, *M* = 1334 ms, *SD* = 502, than to the non-handled objects, *M* = 1119 ms, *SD* = 357. Because the duration of looks that occur later in the trial are potentially constrained by the durations of earlier looks in that trial, we examined the distribution of look lengths and found that 75% of all looks were <1,500 ms in duration. In addition, the duration of looks actually *increased* with increased index (see Figure 3). Thus, there is little evidence that long early looks suppressed the length of later looks.

We performed an LMM with duration of each individual look as the DV as described in the *Analysis Plan* section. This model showed a significant omnibus effects of look index, *F*_(1, 3319.7)_ = 57.75, *p* < 0.001, due to infants' looks increased in duration across the trial, and salience, *F*_(1, 1720.8)_ = 13.42, *p* < 0.001, due to infants' looks increasing with increased object salience.

Importantly, the model revealed a significant interaction between motor level and object type, *F*_(1, 3244.8)_ = 6.37, *p* = 0.012, and a 3-way interaction with these two variables and look index, *F*_(1, 3252.4)_ = 4.17, *p* = 0.041. Thus, motor level was related to the duration of infants' looks at handled versus non-handled objects. The 3-way interaction is displayed in Figure 3. Non-crawling infants (motor levels 1 and 2), demonstrated little or no difference in the duration of looks to handled and non-handled objects (with a suggestion that a difference may emerge in the later looks). For infants who are standing or walking (motor levels 4 or 5), early looks to the handled item are longer than early looks to the non-handled item. Thus, the three-way interaction stems from differences in the timing of when infants with different motor abilities show a preference for the handled item. Again, our analysis with age in days instead of motor development yielded no significant effects of or interactions with age (see Supplemental materials).

## Discussion

We observed that infants' visual object preferences were related to motor development, adding to a growing literature showing connections between motor development and infants' visual object perception and processing (Needham et al., 2002; Sommerville et al., 2005; Libertus and Needham, 2010; Soska et al., 2010). In addition, we successfully measured infants' visual preferences using online tools. These tools can be effective in advancing our understanding of infant cognitive development. Our findings confirm observations made by Scott et al. (2017) that meaningful data can be obtained using online tools, such as Lookit.

Our results are generally consistent with previous literature showing a relation between infants' object perception and motor development (Baumgartner and Oakes, 2013; Kretch et al., 2014; Franchak, 2018). Specifically, we observed that the duration of infants' individual looks to handled vs. non-handled items varied as a function of their motor level. Our results corroborate those of Libertus et al. (2013), who showed that infants with more reaching experience exhibited preferences for more graspable objects. Here we show that the cascading effect of multiple aspects of motor development influence how infants look at objects. Although the focus of previous research has been on reaching experience, achievements such as standing and walking also change infants' attention to, perception of, and interactions with objects (Karasik et al., 2011; Frick and Möhring, 2013). Thus, although our results cannot provide direct insight into why standing and walking would enhance infants' preference for handled objects *per se*, they are consistent with literature showing that object perception and preferences are related to gross motor development.

Of course, because motor level and age were confounded it is impossible to completely disambiguate them; it is possible that infants' increasing interested in handled objects is due to other factors (e.g., cortical maturation, experience). However, our findings suggest that changes in handle preference are due, at least in part, to motor development. First, because motor development increases with age, changes in motor abilities—and the interactions with objects that accompany them—also change with age. Age effects may actually reflect changes in motor development. Second, interactions emerged in our sample when modeling the effect of motor development on infants' looks, but not when modeling the effect of age. Finally, studies using an age-held-constant design, comparing infants of the same age who differed on motor abilities, have observed that motor development is associated with changes in object perception (e.g., Soska et al., 2010; Rakison and Krogh, 2012; Libertus et al., 2013; Ross-Sheehy et al., 2016). Thus, although we cannot completely rule out age effects other than those attributable to motor development, it seems likely that our findings reflect, at least in part, change in motor ability.

Because our data were collected using families' own computers and webcams, it was necessarily more variable in quality than data collected in the lab. However, we demonstrate here that the quality of the data collected allowed for nuanced and in-depth data analysis at the level of infants' individual looks. Although looking coded from video does not have the temporal resolution of eye tracking data, we were nevertheless able to examine infants' behavior at multiple levels, gaining deeper insight into their looking behavior and how their visual preferences ebb and flow over time.

Specifically, our results indicate that infants' preference for the handled object–and differences between infants of different motor levels–occurred at the level of their individual looks. Early looks by more motorically advanced infants, (i.e., those who could crawl, stand, or walk) were longer to handled than to non-handled objects; this difference decreased over time. Thus, the Lookit platform, or other tools for online infant data collection, can generate the quality of data that allows researchers to ask sophisticated questions about the nature of infants' looking behavior and how it changes not only over development, but also from moment to moment during a trial.

Our analytic approach also allowed us to control for various potential confounding variables. Each of our effects of interest were obtained in analyses controlling for differences in object salience and object size. Thus, although infants generally looked longer to more salient objects, the effects of handled vs. non-handled were obtained in analyses that controlled for these potentially confounding factors.

This study was conducted online out of necessity due to the COVID-19 pandemic. However, the results contribute to our growing understanding of how motor development is related to infants' object perception, adding novel findings to the work showing such relations. In addition, we demonstrate how data obtained via online platforms can be effective in conducting sophisticated analyses that provide insight beyond overall preferences for one stimulus over another. Thus, online testing is an important avenue for future research in infant development.

## Data Availability Statement

The data and R scripts are available in this repository: https://osf.io/a4ms6/.

## Ethics Statement

The studies involving human participants were reviewed and approved by UC Davis Institutional Review Board Administration. Written informed consent from the participants' legal guardian/next of kin was not required to participate in this study. Instead, parents provided a video recording of their consent.

## Author's Note

Research materials, statistical analyses, Supplementary Materials, and data sets are available from OSF (https://osf.io/a4ms6/?view_only=8974f5297e25457fa38ee815d7f760fe) and videos of subject sessions are available at Databrary (https://nyu.databrary.org/volume/1316).

## Author Contributions

CN and LO contributed to conception and design of the study. CN collected the data, conducted the data analysis, and wrote the first draft of the manuscript. LO contributed to the data analysis and wrote sections of the manuscript. Both authors contributed to manuscript revision, read, and approved the submitted version.

## Funding

This research and preparation of this manuscript was made possible by NIH grant R01EY030127 awarded to LO.

## Conflict of Interest

The authors declare that the research was conducted in the absence of any commercial or financial relationships that could be construed as a potential conflict of interest.

## Publisher's Note

All claims expressed in this article are solely those of the authors and do not necessarily represent those of their affiliated organizations, or those of the publisher, the editors and the reviewers. Any product that may be evaluated in this article, or claim that may be made by its manufacturer, is not guaranteed or endorsed by the publisher.

## Footnotes

^1^IRB and funding disallowed participation by or compensation for non-US participants.

^2^Variation in framerates resulted from differences in web cameras, upload speed, etc. We used a custom script to extract the framerate from each video and determine the duration of each infants' video frames.

^3^We conducted Spearman's Rank Order Correlations when examining relations with motor level, which was ordinal.
