# Supplementary figures and images for: “May I Grab Your Attention?”: An Investigation Into Infants' Visual Preferences for Handled Objects Using Lookit as an Online Platform for Data Collection
Source: Front Psychol. 2021 Sep 10;12:733218. doi: 10.3389/fpsyg.2021.733218 (PMC8460868; doi:10.3389/fpsyg.2021.733218)

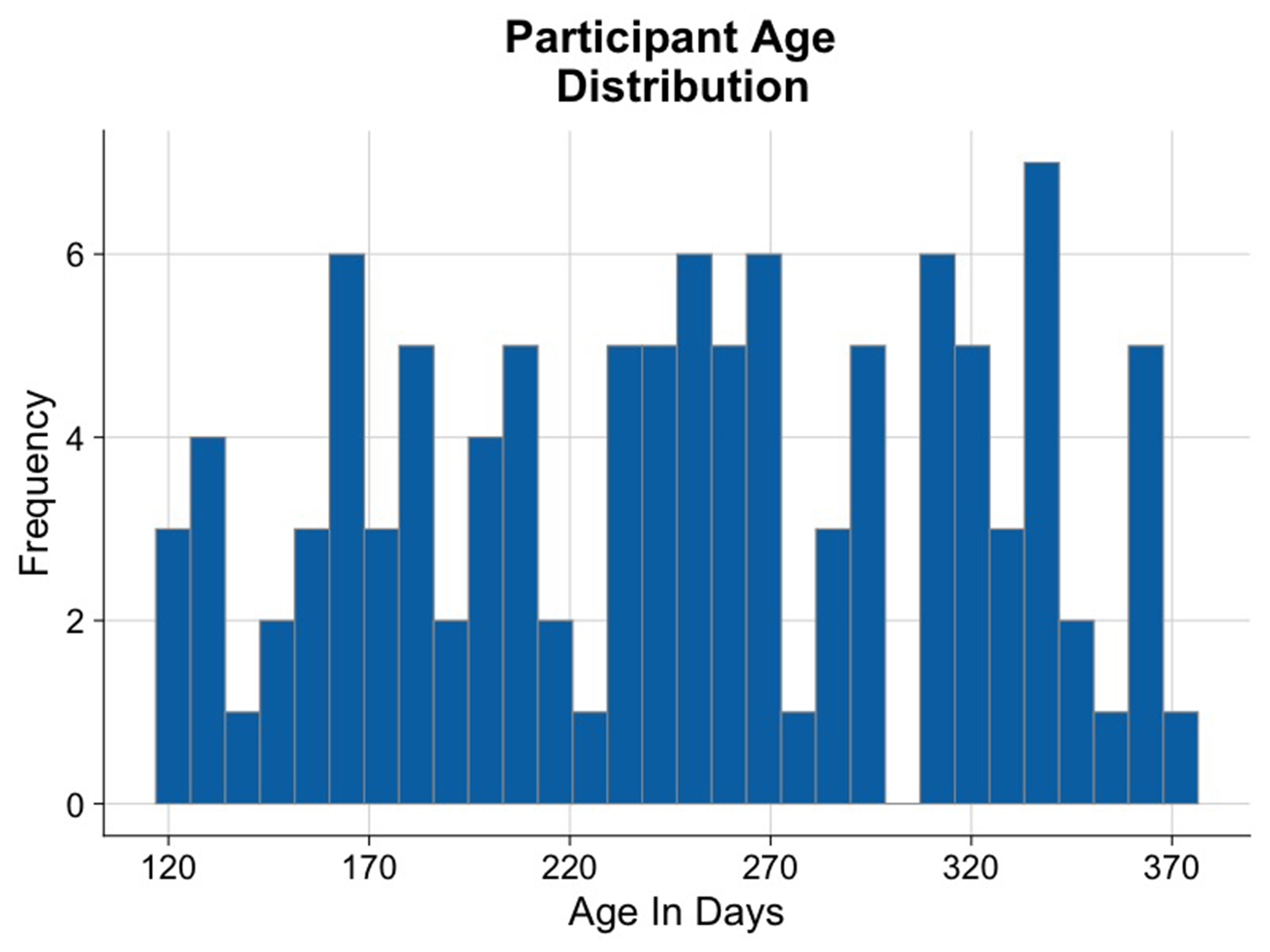

Supplement: Supplementary file 1 [file Image_1.JPEG]
