# Supplementary material for: “May I Grab Your Attention?”: An Investigation Into Infants' Visual Preferences for Handled Objects Using Lookit as an Online Platform for Data Collection
Source: Front Psychol. 2021 Sep 10;12:733218. doi: 10.3389/fpsyg.2021.733218 (PMC8460868; doi:10.3389/fpsyg.2021.733218)
Supplement: Supplementary file 2 [file Table_1.DOCX]

**A**. Linear Mixed Effects Model:

**Motor Level** x **Trial Order** x **Stimulus Salience** x **Stimulus Size**

|  | **Handle Preference** | | |
| --- | --- | --- | --- |
| *Predictors* | *Estimates* | *CI* | *p* |
| (Intercept) | 0.02 | -0.09 – 0.13 | 0.732 |
| Motor Level | 0.01 | -0.02 – 0.04 | 0.392 |
| Trial Order | 0.00 | -0.02 – 0.02 | 0.742 |
| Stimulus Salience | 0.47 | 0.10 – 0.84 | **0.013** |
| Stimulus Size | -0.18 | -0.38 – 0.02 | 0.083 |
| Motor Level * Trial Order | 0.00 | -0.01 – 0.01 | 0.884 |
| SD (Intercept) | 0.03 |  |  |
| SD (Intercept) | 0.07 |  |  |
| SD (Observations) | 0.46 |  |  |
| **Random Effects** | | | |
| σ^2^ | 0.04 | | |
| τ_00_ _child_hashed_id_ | 0.00 | | |
| τ_00_ _filename_ | 0.00 | | |
| ICC | 0.11 | | |
| N _filename_ | 8 | | |
| N _child_hashed_id_ | 107 | | |
| Observations | 721 | | |
| Marginal R^2^ / Conditional R^2^ | 0.084 / 0.182 | | |

B. Linear Mixed Effects Model:

**Age in Days** x **Trial Order** x **Stimulus Salience** x **Stimulus Size**

|  | **Handle Preference** | | |
| --- | --- | --- | --- |
| *Predictors* | *Estimates* | *CI* | *p* |
| (Intercept) | 0.06 | -0.01 – 0.12 | 0.082 |
| Age in Days | 0.01 | -0.02 – 0.05 | 0.455 |
| Trial Order | 0.01 | -0.00 – 0.01 | 0.196 |
| Stimulus Salience | 0.47 | 0.10 – 0.84 | **0.012** |
| Stimulus Size | -0.18 | -0.38 – 0.02 | 0.081 |
| Age in Days * Trial Order | 0.00 | -0.01 – 0.01 | 0.825 |
| SD (Intercept) | 0.03 |  |  |
| SD (Intercept) | 0.07 |  |  |
| SD (Observations) | 0.46 |  |  |
| **Random Effects** | | | |
| σ^2^ | 0.04 | | |
| τ_00_ _child_hashed_id_ | 0.00 | | |
| τ_00_ _filename_ | 0.00 | | |
| ICC | 0.11 | | |
| N _filename_ | 8 | | |
| N _child_hashed_id_ | 107 | | |
| Observations | 721 | | |
| Marginal R^2^ / Conditional R^2^ | 0.084 / 0.181 | | |

C. Linear Mixed Effects Model:

**Motor Level** x **Stimulus (no handle)** x **Fixation Number** x

**Stimulus Salience** x **Stimulus Size**

|  | **Length of Fixation** | | |
| --- | --- | --- | --- |
| *Predictors* | *Estimates* | *CI* | *p* |
| (Intercept) | 422.73 | 4.80 – 840.66 | **0.047** |
| Motor Level | 59.59 | -35.42 – 154.59 | 0.219 |
| Stimulus (no handle) | 118.61 | -222.41 – 459.64 | 0.495 |
| Fixation Number | 217.81 | 150.77 – 284.84 | **<0.001** |
| Stimulus Salience | 1581.71 | 735.52 – 2427.90 | **<0.001** |
| Stimulus Size | -2167.63 | -6028.54 – 1693.27 | 0.271 |
| Motor Level *  Stimulus (no handle) | -129.04 | -229.25 – -28.84 | **0.012** |
| Motor Level * Fixation Number | -24.80 | -44.08 – -5.53 | **0.012** |
| Stimulus (no handle) *  Fixation Number | -61.09 | -153.16 – 30.98 | 0.193 |
| (Motor Level *  Stimulus (no handle)) * Fixation Number | 28.22 | 1.13 – 55.32 | **0.041** |
| SD (Intercept) | 384.06 |  |  |
| SD (Intercept) | 134.49 |  |  |
| SD (Observations) | 29.54 |  |  |
| **Random Effects** | | | |
| σ^2^ | 761847.49 | | |
| τ_00_ _child_hashed_id_ | 147503.89 | | |
| τ_00_ _stim_ | 18086.23 | | |
| ICC | 0.18 | | |
| N _stim_ | 8 | | |
| N _child_hashed_id_ | 107 | | |
| Observations | 3340 | | |
| Marginal R^2^ / Conditional R^2^ | 0.102 / 0.262 | | |

D. Linear Mixed Effects Model:

**Age in Days** x **Stimulus (no handle)** x **Fixation Number** x

**Stimulus Salience** x **Stimulus Size**

|  | **Length of Fixation** | | |
| --- | --- | --- | --- |
| *Predictors* | *Estimates* | *CI* | *p* |
| (Intercept) | 610.50 | 323.04 – 897.95 | **<0.001** |
| Age in Days | 21.61 | -89.30 – 132.52 | 0.703 |
| Stimulus (no handle) | -293.01 | -415.57 – -170.45 | **<0.001** |
| Fixation Number | 138.99 | 116.46 – 161.52 | **<0.001** |
| Stimulus Salience | 1589.64 | 743.36 – 2435.93 | **<0.001** |
| Stimulus Size | -2183.63 | -6008.44 – 1641.18 | 0.263 |
| Age in Days *  Stimulus (no handle) | -106.38 | -223.40 – 10.64 | 0.075 |
| Age in Days * Fixation Number | -26.15 | -48.52 – -3.77 | **0.022** |
| Stimulus (no handle) * Fixation Number | 28.57 | -2.99 – 60.14 | 0.076 |
| (Age in Days * Stimulus (no handle)) * Fixation Number | 19.69 | -11.75 – 51.14 | 0.220 |
| SD (Intercept) | 379.17 |  |  |
| SD (Intercept) | 133.02 |  |  |
| SD (Observations) | 29.55 |  |  |
| **Random Effects** | | | |
| σ^2^ | 762055.11 | | |
| τ_00_ _child_hashed_id_ | 143768.61 | | |
| τ_00_ _stim_ | 17694.63 | | |
| ICC | 0.17 | | |
| N _stim_ | 8 | | |
| N _child_hashed_id_ | 107 | | |
| Observations | 3340 | | |
| Marginal R^2^ / Conditional R^2^ | 0.105 / 0.261 | | |
